# Salt Intake and Health Risk in Climate Change Vulnerable Coastal Bangladesh: What Role Do Beliefs and Practices Play?

**DOI:** 10.1371/journal.pone.0152783

**Published:** 2016-04-04

**Authors:** Sabrina Rasheed, A. K. Siddique, Tamanna Sharmin, A. M. R. Hasan, S. M. A. Hanifi, M. Iqbal, Abbas Bhuiya

**Affiliations:** Centre for Equity and Health Systems, International Centre for Diarrhoeal Disease Research, Bangladesh (icddr,b), Shahid Tajuddin Ahmed Sarani, Mohakhali, Dhaka 1212, Bangladesh; BRAC, BANGLADESH

## Abstract

**Background:**

High salt consumption is an important risk factor of elevated blood pressure. In Bangladesh about 20 million people are at high risk of hypertension due to climate change induced saline intrusion in water. The objective of this study is to assess beliefs, perceptions, and practices associated with salt consumption in coastal Bangladesh.

**Methods:**

The study was conducted in Chakaria, Bangladesh between April-June 2011. It was a cross sectional mixed method study. For the qualitative study 6 focus group discussions, 8 key informant interviews, 60 free listing exercises, 20 ranking exercises and 10 observations were conducted. 400 adults were randomly selected for quantitative survey. For analysis we used SPSS for quantitative data, and Anthropac and Nvivo for qualitative data.

**Results:**

Salt was described as an essential component of food with strong cultural and religious roots. People described both health benefits and risks related to salt intake. The overall risk perception regarding excessive salt consumption was low and respondents believed that the cooking process can render the salt harmless. Respondents were aware that salt is added in many foods even if they do not taste salty but did not recognize that salt can occur naturally in both foods and water.

**Conclusions:**

In the study community people had low awareness of the risks associated with excess salt consumption and salt reduction strategies were not high in their agenda. The easy access to and low cost of salt as well as unrecognised presence of salt in drinking water has created an environment conducive to excess salt consumption. It is important to design general messages related to salt reduction and test tailored strategies especially for those at high risk of hypertension.

## Introduction

High blood pressure is the major risk factor of cardiovascular diseases especially heart diseases and stroke [1]. In South East Asia, an estimated 36% adults have hypertension [2]. In Bangladesh more than 27% of all deaths [3] are thought to be associated with cardiovascular diseases. Epidemiological and experimental studies suggest that high salt consumption is an important risk factor of elevated blood pressure[4,5]. Globally, in 2010, an estimated 1.65 million deaths from cardiovascular diseases were attributed to high salt consumption [6]. Clinical trials and population-based studies have shown that reducing salt intake at the population-level is associated with reduction in rates of hypertension [7,8] and that such strategies are cost effective. However, only a few countries have implemented this successfully as a public health intervention [9].

The source of dietary salt intake varies among different population groups depending on whether substantial amounts of processed foods were consumed or foods were essentially prepared at home, sources of drinking water and cooking methods[10,11]. In developed countries, people take sodium in the diet mainly through processed foods [12]. In contrast, the source of dietary salt in Japan and China was food cooked with soy sauce and fish sauce[8]. Behaviour around food preparation and salt use are constructs of historical, social and cultural factors [13,14]. As cultural construction, food consumption is dictated by beliefs and customs around types of food to be eaten, food preparation, vocabulary and values attached to food, and beliefs around how foods affect health. It is imperative that we learn about lay cultural constructs that prevail among communities to design effective strategies to reduce population-level salt consumption among people at risk of hypertension.

Bangladesh is a low-lying land mass comprising the delta of the Ganges and Brahmaputra rivers. The coastal zone, which includes exposed coast (areas located in the estuary or facing the Bay) and internal coast (areas behind the exposed coast) covers approximately 47,000 square kilometer of land area. This is nearly 32% of total landmass of the country with approximately 48 million people living in the coastal area [15]. Climate change induced increase in Sea Surface Temperature causes increased frequency of cyclonic events and storm surges and as well as sea level rise are resulting in intrusion of saline water in the coastal belt of Bangladesh [16]. People of those areas who rely on rivers, ground water and ponds for drinking water have a higher level of salt intake [17,18] than those living more inland that may increase their risk of hypertension [18,19]. Salt intake through drinking water could result in about 1.1g/day of sodium intake according to a calculation based on mean sodium levels in drinking water [20]. Furthermore, the availability of locally produced cheap salt in the area may also affect salt use in this population[21].

The World Health Organization (WHO) recommends the consumption of a maximum of 5g salt/day for adults[22]. However, according to the INTERSALT [23] study the average consumption of salt in 52 study sites ranged between 5.9–11.7g/day. This was far in excess of what is recommended. There are no national surveys of assessment of population-level salt consumption in Bangladesh. However, two studies conducted in coastal locations—one among pregnant women and the other among healthy adult population reported a mean consumption of 9g/day and 6g/day respectively [17,18] which is in excess of the maximum levels recommended.

The objective of this study is to explore the sources of dietary salt, assess beliefs, perceptions, and practices associated with salt consumption particularly, among the coastal population of Bangladesh who are likely at high risk of hypertension due to exposure to environmental salinity.

## Methods

### Study area

The study was conducted in Chakaria (sub district)-a rural area of the Southeastern coastal region of Bangladesh during April-June 2011. Chakaria has an existing health and demographic surveillance system (HDSS) run by Centre for Diarrhoeal Disease Research, Bangladesh (icddr,b) and a quarterly survey of health and demographic events are collected through the HDSS. The total HDSS area is about 288 KM^2^. About half of the HDSS area belongs to low-lying coastal area and the rest of the area has plain land and hilly area. Availability of the HDSS allowed us to sample respondents for our quantitative study. Details of the study area and the HDSS have been provided elsewhere [24].

### Study design

The study was a cross sectional in design where both qualitative and quantitative data collection techniques were deployed. For qualitative data, six qualitative methods were used- key informant interviews (KII), focus group discussion (FGD), free listing, ranking and observation (Table 1). The respondents were selected through purposive homogeneous sampling to encapsulate a wide range of perspectives regarding the use of salt. Some criteria used for selecting respondents were gender and age.

**Table 1:** Methods used and sample size.

Methods	Types of Respondents	Number
KII	Village doctor, religious leaders, teachers (School and college)	8
FGD	Small grocery shop owner, small restaurant owner, member of hypertension club, housewives and college students (Male/ female), adult males and females	6
Free listing		60
Ranking exercise		20
Observation	Eating behaviour within and outside the house	10

At the preparatory stage free listing [25] was used to lists food where salt is added. The names of foods that were listed were written on small cards for ranking exercise. The respondents were then asked to rank the foods in the order of preference. Observation, FGD, and KII were performed to understand community perception regarding salty food, salt consumption, and the practice of salt use. The FGDs and KIIs were conducted in the time and place chosen by the respondents. The KII took 45 minutes to an hour while the FGDs took about 1.5 hours on average. All the KIIs and FGDs were audio-recorded and transcribed in Bangla for analysis by the researchers themselves. Extensive field notes were maintained during field work to supplement the data collected. The qualitative field team consisted of 2 male and 2 female researchers with a Masters degree in Anthropology and social science, as well as 4-5years of experience in collecting qualitative data. All the researchers who collected data were trained by a medical anthropologist (TS) and a public health specialist (SR). Among those who collected data 2 are authors of this paper (TS and AH). Qualitative study results were used to form the quantitative survey instrument.

For the quantitative study to calculate the sample size we assumed that 50% of the population will consume more that 5g of salt/day. Based on this assumption and considering a 5% refusal to participate we needed 403 people for the survey. From the list provided by HDSS 609 people were approached, 421 people were available, 15 were excluded and finally data from 400 respondents over 18 years of age was available for analysis. The respondents were randomly selected from 5 villages representing coastal area, plain land and hilly area. The respondents were approached for a survey, anthropometric data and collection of 24 hours urine output (not reported in this paper). Data was collected on perceptions regarding the risk of hypertension, behaviour and risk perception regarding salt use and frequency of eating salty foods identified during listing and ranking exercises. Details of the methodology of the quantitative survey are provided elsewhere [17]. For this paper quantitative data was used to support the qualitative insights.

All the participants of the study provided written consent and the study received ethical clearance from the Ethical Review Committee of International Centre for Diarrhoeal Disease Research, Bangladesh (icddr,b). During data collection, digital recorder was used to record the conversation after ensuring consent.

### Data analysis

**Fig 1:**
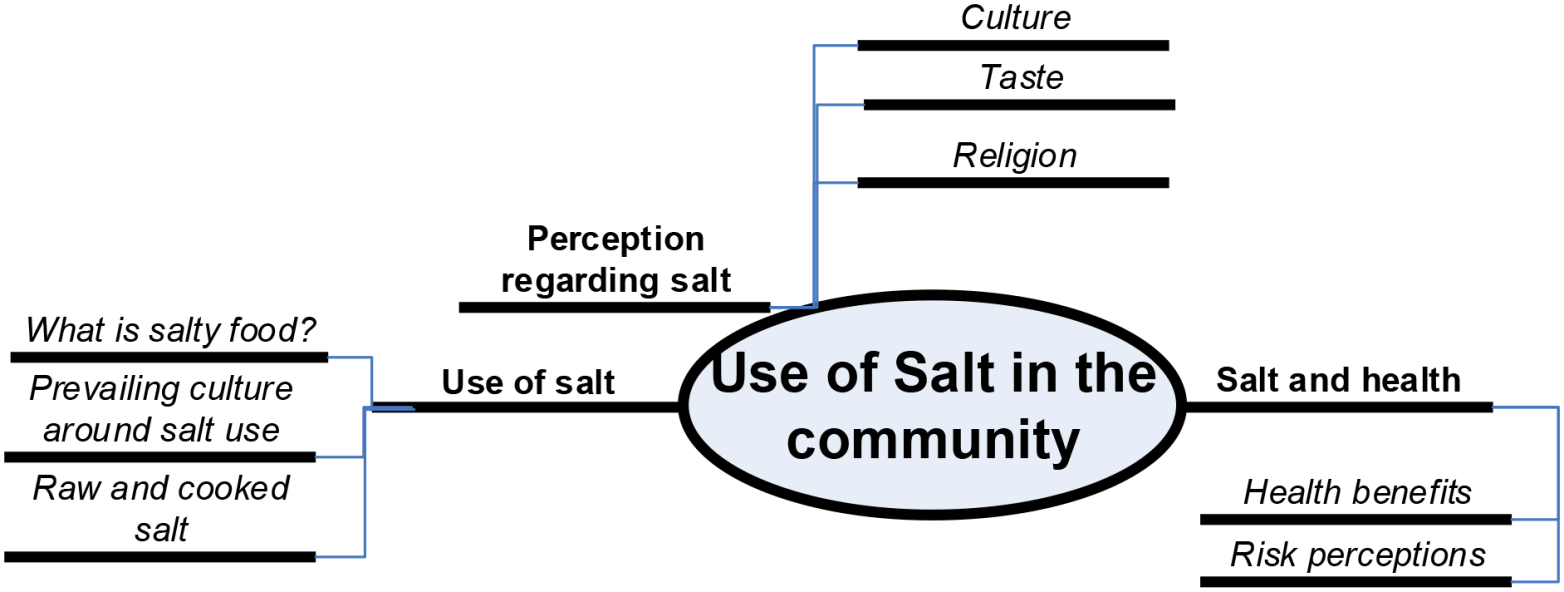
Code tree.

Data from free listing and ranking exercise was analyzed with computer software ANTHROPAC (Borgatti, 1996). The data obtained was coded for emerging themes using Nvivo (Fig 1). Constant comparative analysis was conducted to come up with the sub codes. The emergent themes were triangulated using data collected through both FGDs and KIIs. Peer debriefing was conducted within the study team to understand the issues and consolidate the findings.

The quantitative data was analyzed using SPSS version 12. Descriptive statistics were generated from the quantitative survey to support the qualitative analysis. The meaning of the phrases used throughout the paper is provided for clarification in Table 2.

**Table 2:** Phrases used and their explanations.

Phrases used in the paper	Definitions
*Based on processing of salt*	
a)Refined salt	Salt that was processed in a factory
b)Iodized salt	Salt processed in a factory and iodine added to it
c)Unrefined salt or *khola lobon*	Salt locally produced from sea water but did not go through factory processing
*Based on the use of salt*	
a)Cooked salt	Salt that was used during cooking or which went through some cooking process
b)Table salt	The salt that was added to prepared food

## Results

### Respondent profile

The FGD respondents were mostly between 20–39 years of age. FGDs were conducted separately for males and females. Female respondents were mostly housewives. All the key informants were males and were older and better educated than the respondents in the FGDs (Table 3).

**Table 3:** Characteristics of respondents in the qualitative study.

Characteristics	Focus group discussion (n = 6)	Key informant interview(n = 8)
***Age group***		
20–29	20	0
30–39	11	3
40–49	8	3
50–59	4	2
***Gender***		
Male	25	8
Female	18	0
***Profession***		
House wife	6	0
Grocery shop owner	6	0
Small restaurant owner	6	0
College student	18	0
Hypertension club member	7	0
Teacher	0	2
Village doctor	0	3
Religious leader	0	3
***Years of schooling***		
0–5	20	0
6–10	5	3
10+	18	5

### Context

In Chakaria many farmers are involved in salt production from sea water during dry season. The locally produced unrefined salt is bought by the salt refineries, salt vendors (for local market) and villagers (for household consumption). During the dry season villagers purchase enough unrefined salt to last a year from the farmers. Salt farmers also keep enough salt for yearly household consumption and often supply relatives with the *khola lobon* (unrefined salt). The unrefined salt is priced at 3 to 4 taka/kg during production season and 5–6 taka/ kg during off season. Along with unrefined salt both refined iodized and refined non-iodized salt are available in the local market. A kg of refined iodized salt costs between 20–25 taka whereas refined non-iodized costs between 10–12 taka. Mostly people use that *khola lobon* (unrefined salt) in food preparation.

### Perceptions regarding salt

#### Taste

Respondents explained that salt was an integral element of food and the taste enhancing property of salt is the reason why salt is used in food. The respondents’ usual diet consisted of rice (*Bhaat*) and curry which is often flavored with oil, spices, chilli and salt. Absence of chilli, spice and oil from curry was considered acceptable but not the absence of salt. One respondent perceived expensive foods as ‘tasteless’ and ‘useless’ in absence of adequate salt. The amount of salt used in food preparation depends on individual preference. According to respondents-

*Food without salt is like a pond without water*, *a woman without modesty and a cloud that doesn’t produce rain*. *Food without salt does not satisfy*.

(KII-3)

Salt enhanced appetite according to respondents. Old people who lacked appetite and those who could not taste food due to their smoking or betel nut chewing habits, added extra salt to food to make it palatable. As one woman respondent explained-

*Usually my husband is not happy with same amount of salt as we normally use in our family meal*. *He (as an old person) has problems tasting (food)… so he takes extra salt with bhaat (rice) to make it tasty*.

(FGD-2)

Often mothers added salt to rice for their children. One mother explained that when children did not like to eat rice and curry, adding salt to rice allowed them to eat during meals. Often salt was added to sweet snacks such as S*uzi*, *Semai* and *Halua*, which are traditional infant foods to enhance the sweetness of the food.

#### Culture and religious perspective

In Chakaria taking table salt on the plate with a meal is a common practice. According to the respondents people often started their meal with rice and salt and different curries were added to the rice afterwards. During meal time a salt pot is commonly provided with the food and people took one or two spoonful of salt on their plates before starting a meal. As stated by a male respondent-

*All the members of my family start meals with rice and salt*. *Even when we are invited (outside the house)*, *we ask for Aalga lobon (table salt) if the host did not provide (it)*. *In most places host provides extra salt on a plate or pot (during the meal)…*..

(KII 6)

People spoke about acquiring this habit from their parents.

As one respondent said:

*I saw my parents and grandfather taking alga lobon (table salt) with food*. *I learnt from them and my son is also learning*.

(FGD-2)

During KIIs and FGDs respondents talked about poor people using chilli and salt to make rice edible when they could not afford vegetables or fish. Taking extra salt with a local delicacy called *Panta bhaat* (leftover rice fermented with water) was also very popular in this area.

Religious leaders and elderly people mentioned a Muslim religious instruction about eating salt.

*Taking a little salt prior to a meal is Sunnah(the way of life prescribed by the prophet Muhammad)*. *Our Prophet used to take alga lobon(table salt)before starting and after finishing his meals*. *It is in the Hadith and it is our duty to follow (the rule)*.

(KII-6)

### Use of salt in food

#### What are salty foods?

Respondents said that most foods were prepared with some salt in them although they may not taste salty. None talked about the natural existence of salt in food or water. When specifically probed about salt in drinking water respondents spoke about drinking water tasting different in different areas but did not link the taste specifically to salt. Salty foods were considered the tastiest but adding salt to sweet and sour foods were also widely practiced. During free listing exercises, respondents mentioned different food they ate and their source of salt (Table 4). In addition to the many salty foods where salt was added during preparation, they added table salt to rice and salad (mainly made of cucumber) during meals. Salt was also added to sweet and sour fresh fruits such as *kathal* (jackfruit) *kacha aam* (green mango), *boroi* (plum) and *tetul*(tamarind) to enhance sweetness or reduce sourness. Some bland fruits such as *bangi* (rock melon) and *khira* (cucumber) needed salt to make them tasty.

**Table 4:** Foods consumed in the community and when salt was added to them.

Salt added during food preparation at home	Table salt added to food	Available in prepared food bought outside home
-Curry	-Rice, *Muri* (puffed rice)	-Drinks: Soft drinks, fruit juice
-Fish: *Samudrik maach* (sea fish), salted hilsa, *Shutkimaach* (dried fish)	-Sour fruits: K*acha aam*(green mango), *boroi* (plum), *tetul* (tamarind)	-Packed snacks: Biscuit,chips, *chanachur*, fruit juice
-*Pitha*(Rice cake)	-Salad	-Breads: butter bun, plain bread, *naan*, *roti*, *paratha*
-Sweet dishes: *semai* (vermicelli), *suji* (semolina), firni (rice pudding), payesh	-Melon: Watermelon, *Bangi*	-Locally prepared snacks: s*hingara*,*shomucha*,*dalpuri*, *piaju*, *chhola*
-Noodles	-Sweet fruits: Pineapple, jackfruit	
-Pickles	-Nuts	
-*Khichuri* (mix of rice and lentil)		
-Drink: s*horbot* (lemonade), Milk, Tea		
-Yoghurt		

#### Prevailing culture around using salt

According to the quantitative survey results, cooked food was the major source of salt in their diets and unrefined salt was often used for both cooking and as table salt (Table 4). Respondents added extra salt to their meals (26%) as well as to fresh fruits (78%). Table salt was mostly added to food to enhance taste as well as due to habit. Very few took added salt for health benefit (Table 5).

**Table 5:** Salt use patterns among respondents.

Variables	Proportion (%)
***Main source of salt (n = 394)***	
Cooked food	89.6
Table salt	10.1
Don't know	0.3
***Type of salt used in cooking (n = 395)***	
Unrefined	91.4
Refined non-iodized	1.5
Refined iodized	7.1
***Type of salt used as table salt (n = 395)***	
Unrefined	83.3
Refined non-iodized	4.9
Refined iodized	10.8
***Taking table salt with food (n = 395)***	
Yes	25.8
***Taking table salt with fruits (n = 395)***	
Yes	78.4
***Reason for taking table salt with food (n = 102)***	
Enhance taste	81.2
Health benefit	3.0
Habit	10.3
Don't know	5.0

#### Refined and unrefined salt

The qualitative inquiry revealed that availability and low price of *khola lobon* (unrefined salt) played an important role in its preference. As two respondents stated-

*“A day labourer earns only three hundred taka daily which is not enough to buy everything he requires*. *If he buys rice*, *then he cannot buy fish or vegetables*. *So he has to buy khola lobon (unrefined salt) which is cheap”*

(FGD-3)

*“A family in our area only spends 200 taka for salt (unrefined salt) and that is enough for one year*. *But if that family buys iodized salt with 200 taka then they would need to buy more after two months*. *Then why would they buy other (iodized)salt*?*”*

(FGD-5)

As unrefined salt had been used for cooking for many years in this community, the cooking style and recipes developed with unrefined salt in mind. As one respondent said very eloquently-

*“A woman learns from her mother and grandmother who use* *khola lobon* *(unrefined salt) in cooking*. *She can easily measure how much salt is needed for a particular dish*. *It is easier to measure large granule salt (unrefined salt) rather than small granule salt (refined salt)*

(KII-7)

In addition to being used to using unrefined salt, a few female respondents perceived that unrefined salt was more salty and therefore, less is needed compared to iodized salt. Their opinion was based on their experience of using different kinds of salt.

According to the shop owners interviewed people in the area perceived unrefined salt as being dirty based on its appearance (looked brown or black) and thought that refined salt was clean. Therefore, the restaurant owners said that they used refined non-iodized salt for cooking and as table salt as it is clean but less expensive than iodized salt. Respondents from the community also talked about not preferring unrefined salt as table salt although they used it because of low cost.

*Granule of khola (unrefined salt) is too large and hard*. *On the other hand*, *iodized salt is refined with small granule*. *For this reason*, *we sometimes*, *like to take packet salt (as table salt) with rice*.

(FGD-5)

*We like iodized salt as alga lobon(table salt)because it promptly mixes with rice*.

(FGD-4)

Our observation reflected community preference as we found that unrefined salt was widely used to cook foods while refined non-iodized salt were given as table salt in the small restaurants situated in village markets or to guests at home.

According to one key informant, people who are educated and well off were aware of benefits of iodine and bought iodized salt. Use of iodized salt is seen as sign of prestige in the community as it demonstrated how rich and educated the family was. In some households guests were provided iodized salt as table salt although the family did not use it regularly.

*Many families only keep iodized salt only for showing off (to their guest)*. *It is a kind of* *vodrota* *(courtesy/manners) but they do not use it (regularly)*.

(FGD-5)

### Salt and health

Most of the participants thought that salt was beneficial for health and did not cause any harm. Those who perceived salt as harmful for health only mentioned table salt rather than salt used for cooking. A few respondents thought that table salt is harmful for those with specific illnesses and not everyone.

#### Health benefits of salt

Participants mentioned several perceived health benefit of salt consumption such as purifying blood, cleaning germs and worms from the body, keeping the body in equilibrium and reducing high blood pressure. As one participant said-

*Inside our body*, *there is flow of salted and sweetened water which keeps our body fit to exist*. *This (flow) prevents (pocha) decomposition and (khoy) decay (of our body)*. *Body collects sweet and salt water from food*.

(KII-6)”

Respondents thought that salt has many medicinal properties. Those who were educated linked salt with dehydration. They thought that salt consumption prevents dehydration which can kill a person. When people get dehydrated, they drink oral rehydration solution (ORS) which has salt as an essential component. Some respondents believed that salt can help reduce fat in the body and so many middle aged obese people take table salt with rice in the hope of reducing the extra fat in their body. However, they were not certain how salt reduces fat. A few people mentioned that salt kills germs and worms in the body. Respondents also spoke about using salt to treat abdominal pain and nausea. One respondent described a common practice in the community-

*Sometimes people who suffer from abdominal pain or disturbance (stomach) and vomiting*, *use a mixture of salt with* *chun* *(lime) and* *lebu**(lemon) as a home remedy*. *They mix* *chun* *(lime) and* *lebu**(lemon) with salt water……people get cured immediately*

(KII-4)

#### Risk perceptions

During qualitative exploration some participants mentioned *Aalga lobon*(table salt) with foods causes health problem for pregnant and lactating mothers. One housewife gave an example of a pregnant woman whose legs and feet swelled up because she had a habit of taking table salt with rice. In the similar vein a village doctor mentioned that pregnant women's lives could be at risk if they had pre-eclampsia or eclampsia as a result of taking table salt. The village doctor said that table salt intake among pregnant women increases blood pressure and this leads to eclampsia. The village doctor also advised pregnant or lactating mothers to abstain from taking table salt with meals when they came to seek health care. A few respondents also mentioned that table salt intake can reduce breast milk production among lactating mothers.

*Elderly people of our area always say that it is not good to give table salt to the lactating mother*. *Our elderly people advice lactating mothers not to take table salt as there is possibility of it reducing milk supply*.

(KII-8)

Among the respondents, two people who were members of the local hypertension club (a club formed locally for those who have hypertension with technical assistance from icddr,b and a local paramedic) linked table salt consumption with hypertension. It is important to mention that they believed salt itself is not harmful for health but for those with hypertension, diabetes or kidney diseases, table salt intake was harmful. As a mitigating strategy they often stopped taking table salt with meals. None of the hypertension club members mentioned reducing salt during cooking.

*We had habit of alga lobon(table salt) with bhaat (rice)but now we have given up it*, *brothers(health worker of hypertension club) taught us about the detriment of Aalga lobon(table salt)*

(FGD-2)

For older community members existing religious and cultural beliefs were barriers for reducing salt consumption.

My father is a hypertension patient and he aware that adding salt is harmful for him but he always take Aalga lobon(table salt) while eating bhaat (rice) as Sunnah

(KII-5)

**Fig 2:**
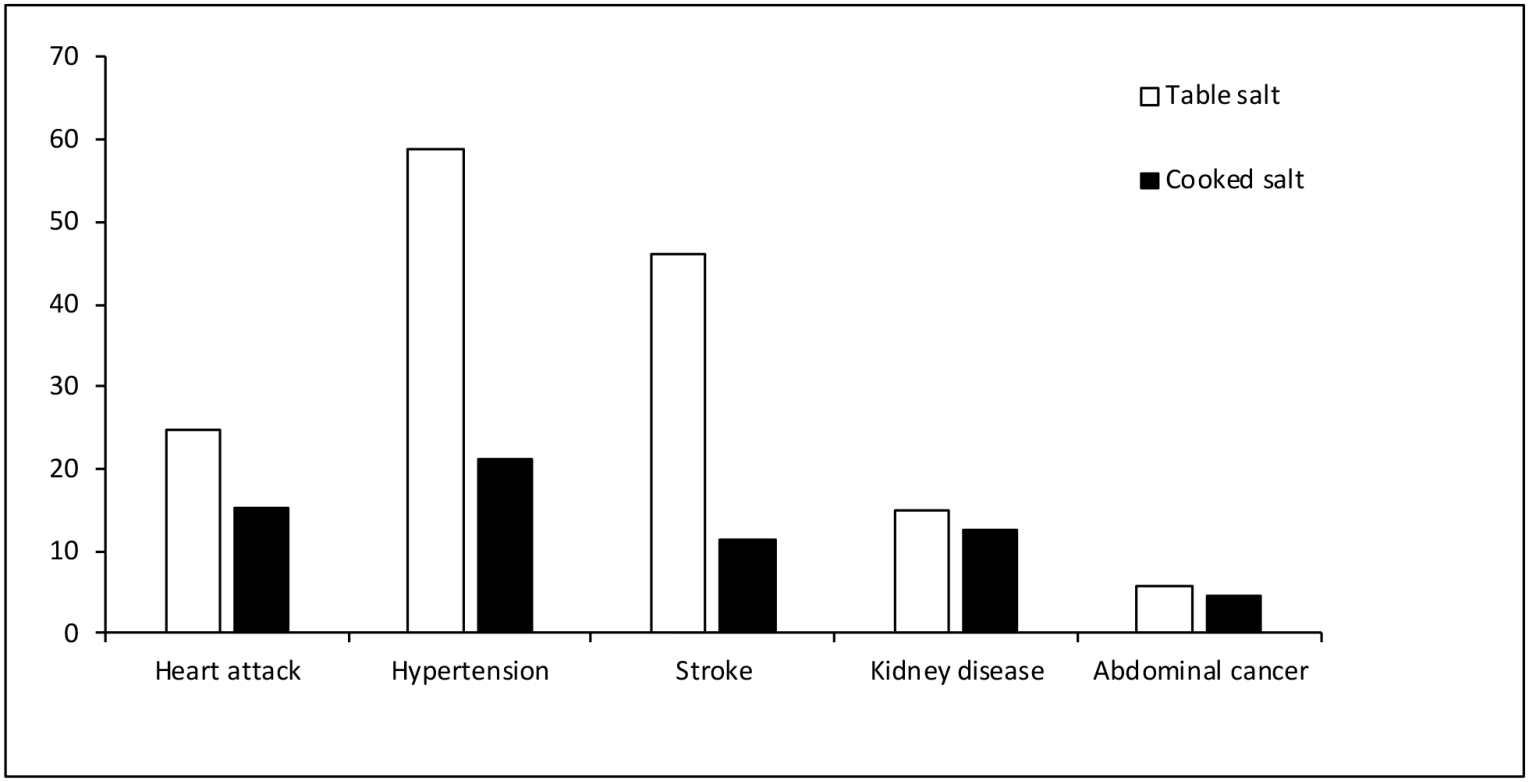
Risk perceptions around table salt and salt used in cooking (n = 394).

During the quantitative survey respondents were asked about different health problems and their link to salt intake. In general risk perception regarding salt intake was low in the population (Fig 2). Respondents linked cardiovascular diseases with excessive salt compared to other diseases. However, those who associated salt intake with different health problems tended to consider table salt more harmful compared to salt used in cooking (Fig 2). This result reflected findings from the qualitative study. Respondents who knew that salt was linked to health problems believed that *Aalga lobon* (table salt) is harmful for the hypertension patients. So respondents who were advised to reduce salt intake by their healthcare providers used more salt during cooking food instead of taking table salt with rice. A few people also mentioned toasting or frying the salt to render it harmless and then using it as table salt.

## Discussion

Our study revealed that salt is as an essential component of food with strong cultural and religious roots. The deep rooted values and meanings associated with use of salt in food indicate that implementation of salt reduction strategies in our study community will be a challenge. In the study community people described both health benefits and risks related to salt intake. Some of the positive perceptions have been influenced by public health promotion of iodized salt and oral rehydration solution. The few negative perceptions were also influenced by preventive messages from the health care providers for those with hypertension. The cultural perspectives such as beliefs and practices presented in this study have, to our knowledge, never been previously reported in Bangladesh. Understanding the existing perceptions and beliefs that affect the patterns of salt use by the community will contribute in designing strategies for reduction of salt intake at community-level.

Historically, salt has played an important role in human society. The value placed on salt is evident by its use in religious ceremonies, cuisines and premise to conquer land [26]. In the Indian subcontinent, introduction of salt taxes led to the movement against British colonial rule that culminated in independence of India in 1947 [27]. Respondents in our study described the religious and cultural significance of salt in their community. Other researchers have reported that foods are often part of the cultural identity of a region and community[28]. The availability and accessibility of salt in our study area may have influenced the evolution of food culture. Similar findings have been shown in others studies conducted in Bangladesh and elsewhere [21,29,30]. Our respondents stated that one of the primary functions of salt is enhancing the flavor of food. Researchers have shown that despite people being used to a certain taste of food, salt used can be reduced about 30% - 50% in composite dishes without significantly reducing acceptability of the dish[31,32]. In contrast others have mentioned that people need time to get used to low salt food[33]. In our study there are some indications that the beliefs about benefits of salt were influenced by knowledge derived from public health activities [34,35]. This demonstrates that individual and cultural beliefs around food, including salt is a dynamic process and can change based on personal experience and exposure to biomedical knowledge[36]. To craft future salt reduction strategies it is important to consider both beneficial and harmful aspects of salt and how to convey this information for maximum benefit to population health.

It is now known that excess salt consumption is associated with various cardiovascular and other diseases [37–40]. Excess sodium intake promotes left ventricular hypertrophy as well as fibrosis in the heart, kidney, and arteries [41–43]. In our study area people at large did not recognize that excess salt consumption is harmful which reflects inadequate biomedical knowledge. However, some of the people in this community knew that salt intake is linked to hypertension indicating that biomedical knowledge of this link, in a limited way, has reached the community. Despite having the knowledge of the link between salt intake and hypertension, people in our study believed that cooking processes can render salt harmless to health. This is an example of medical knowledge being interpreted by people to validate their pre-existing ideas about salt intake, health and illness[36,44]. It also could be that people prioritize taste over knowledge of detrimental effects of food in selecting foods to eat as found in other studies[45]. Most theories of health behavior propose that people who have a good understanding of biomedical knowledge tend to take action to use the knowledge to remain healthy[46]. It is vital, therefore, that recommendations about salt reduction are designed with the understanding of existing cultural beliefs so that people can clearly understand the recommendations and potential benefits associated with them.

One particular important issue to consider is people's awareness about salt content in their foods. For instance, in developed countries researchers reported that many people did not recognize the "hidden" presence of salt in processed foods [9]. In contrast, our respondents were aware that salt is added in many foods even if they do not taste salty. The difference in the findings reflects the differing context because in our study area most foods were prepared at home and people were mostly aware of what has been added to their food. This attribute is largely lost in societies eating a preponderance of processed food. But there is an analog in the population we studied to Western unawareness of salt in processed foods: In our study area, respondents did not recognize that salt can occur naturally in both foods and water. Due to global warming and climate change induced saline intrusion, salinity in drinking water and foods may have already surpassed permissible levels in the exposed coasts of Bangladesh [20] which is becoming an increasingly important health issue. Salt intake through drinking water could result in about 1.1g/day of sodium intake according to a calculation based on mean sodium levels in drinking water [20]. It is important therefore, that in coastal areas with high levels of saline intrusion in water, communication strategies include information about naturally occurring salt in water and food so that people can take that into consideration.

According to the recent Bangladesh national survey (2011) 19.4% males and 31.9% of females over 35 years of age are hypertensive[47] and this trend is expected grow. Climate change induced saline intrusion has already affected the lives over 20 million people living in the exposed coast of Bangladesh[48,49], which may be responsible for the significantly higher proportion of hypertension observed among people living in the coastal area compared to those living in other areas[17,47]. It is imperative therefore, to formulate policies and specific programs in the coastal areas focused on reducing population-level salt intake. It is also important to formulate campaigns to increase consumer awareness of the link between salt intake and health problems[50,51]. The modifiable behavioural factors which also influence increased salt consumption should be given importance in designing tailored salt reduction strategies.

The strength of our study was the use of both qualitative and quantitative methodologies to assess the use of salt from the community perspectives. While qualitative enquiry provided us with a rich description of the context in which salt consumption took place in Chakaria, the quantitative data provided some understanding of the dimension of the problem. In terms of the limitations, our study was conducted in Chakaria and does not represent the entire population of Bangladesh. However, some of the findings related to access and use of salt may be applicable for other coastal areas in Bangladesh and elsewhere.

## Conclusions

In summary, the people in the study community had low awareness of the risk of excess salt consumption and salt reduction strategies were not high in their agenda. With easy access and low cost of unrefined salt and cultural preference for salt consumption, the unrecognized presence of salt in drinking water can push the salt consumption in this population to an unacceptable and unsafe level. Such excessive salt intake can in turn put a large number of people such as pregnant women at risk of hypertension and even death. While it is imperative that awareness raising campaigns are designed for the coastal areas such as Chakaria, given the intractable nature of much of human behaviour it is important to design and test tailored strategies of salt reduction for those at higher risk of hypertension. As there are very strong cultural and religious influences on dietary salt use, and therefore, reduction might be difficult: lowering salt in drinking water (e.g. through offering alternative fresh water sources) would therefore, also be a promising solution to look into. In terms of future research it is important that the effect of environmental salinity on human health is studied over time. With the focus on building resilience against the health effects of climate change articulated within the overall goal of Universal Health Coverage, the findings from our study was of critical importance.
